# Effectiveness of Diphtheria and Tetanus Toxoids and Acellular Pertussis Vaccine in Children Aged 6 Months–6 Years During a Statewide Pertussis Outbreak — Alaska, May–November 2024

**DOI:** 10.15585/mmwr.mm7540a1

**Published:** 2026-10-15

**Authors:** Katherine Newell, Elizabeth Ohlsen, Kristen Moore, Sarah Aho, Louisa Castrodale, Joseph McLaughlin

**Affiliations:** ^1^Section of Epidemiology, State of Alaska Department of Health, Anchorage, Alaska; ^2^Career Epidemiology Field Officer Program, CDC.

**Summary**

**What is already known about this topic?**

After a period of low U.S. pertussis incidence during and immediately after the COVID-19 pandemic, incidence increased in 2024.

**What is added by this report?**

During a 2024 statewide Alaska pertussis outbreak, a matched case-control analysis found that unvaccinated children aged 6 months–6 years had approximately 26 times the odds of having laboratory-confirmed pertussis compared with children who had completed the 3-dose diphtheria and tetanus toxoids and acellular pertussis (DTaP) primary vaccination series ≥2 weeks before specimen collection. The overall adjusted vaccine effectiveness among children who had received ≥3 DTaP doses was 96.2%.

**What are the implications for public health practice?**

Pertussis vaccination is effective in preventing laboratory-confirmed pertussis in young children. These real-world effectiveness findings support adherence to published pertussis vaccination recommendations to ensure strong protection.

## Abstract

Widespread use of pertussis vaccines in children has resulted in a >90% reduction in the number of annual pertussis cases in the United States. After a period of low U.S. pertussis incidence during and shortly after the COVID-19 pandemic, incidence increased in 2024. Data on the real-world effectiveness of diphtheria and tetanus toxoids and acellular pertussis vaccine (DTaP) against pertussis during this disease resurgence are limited. In response to documentation of a record high pertussis incidence in Alaska during May–November 2024, DTaP vaccine effectiveness (VE) against pertussis in Alaska children aged 6 months–6 years was evaluated. Adjusted VE was 96.2% among children who had completed the 3-dose primary DTaP series and 85.3% among those who were partially vaccinated. Compared with children who had received ≥3 DTaP doses, the odds of laboratory-confirmed pertussis were approximately 26 times as high in unvaccinated children (adjusted odds ratio [OR] = 26.1), and approximately 4 times as high in those who had received 1–2 DTaP doses (adjusted OR = 3.8). These findings reinforce the importance of following pertussis vaccination recommendations to protect for children from the disease and reduce the occurrence of pertussis outbreaks.

## Introduction

Pertussis is a highly contagious, vaccine-preventable respiratory bacterial infection caused by *Bordetella pertussis*. Before whole-cell pertussis vaccination began in 1948, an average of approximately 175,000 cases of pertussis were reported among U.S. children per year. Acellular pertussis vaccine (combined with diphtheria and tetanus toxoids as diphtheria and tetanus toxoids and acellular pertussis vaccine [DTaP]) has been recommended for use in infants in the United States since 1997 (*1*). After vaccine introduction, the number of annual pertussis cases in the United States has declined >90% compared with the prevaccine era.

During the COVID-19 pandemic (2020–2023), pertussis incidence in Alaska was unusually low (0.27 cases per 100,000 population in 2022); however, cases surged in 2024, with a record high statewide incidence of 83 cases per 100,000 population. During 2022–2023, estimated DTaP vaccination coverage among young children in Alaska was below the national average; national coverage estimates had also declined before the COVID-19 pandemic (*2*). In Alaska, factors contributing to a resurgence in pertussis incidence might include waning of vaccine-induced and infection-conferred immunity, declining vaccination coverage, heightened clinician awareness and testing, and improved diagnostic capabilities (*2*,*3*). In response to the sharp increase in pertussis cases in Alaska, real-world effectiveness of acellular pertussis vaccine was assessed in children during a statewide pertussis outbreak during May–November 2024.

## Methods

### Data Source and Study Design

Pertussis cases were identified through Alaska’s disease surveillance system, which records all pertussis cases reported to the Alaska Division of Public Health. A matched case-control analysis assessed the association between completion of the primary 3-dose DTaP series and acquisition of laboratory-confirmed pertussis.

### Case Definitions

Case-patients were defined as children aged 6 months–6 years with first-time, laboratory-confirmed pertussis, reported in Alaska, with symptom onset during May 1–November 30, 2024. Case-patients were classified according to the Council of State and Territorial Epidemiologists’ case definition for confirmed pertussis, which includes an acute cough with either isolation of *B. pertussis* or a positive polymerase chain reaction test result.

Control patients were children with acute cough who received testing during the same period for respiratory viral pathogens, but who received a negative pertussis test result or did not receive testing for pertussis. For each case-patient, three control patients were selected using simple random sampling without replacement, matched by birth year and health care facility. To minimize misclassification of disease-conferred immunity as vaccine-induced immunity, children with a documented history of previous pertussis disease were excluded before matching case-patients and control patients; children not born in Alaska were also excluded because immunizations administered outside of Alaska do not appear in Alaska’s immunization information system. If at least one, but fewer than three control patients for a case-patient were available, any who were available were selected; if no control patients were available, the case-patient was excluded. Data on symptom severity and disease outcome were not available and therefore could not be considered when identifying case-patients and control patients.

### Pertussis Vaccination Status

Pertussis vaccination status for both case-patients and control patients was ascertained using VacTrAK, Alaska’s immunization information system; all vaccinations administered in Alaska are required to be reported to VacTrAK. Participants were classified as fully vaccinated if they had received ≥3 valid DTaP doses according to the 2018 recommendations of the Advisory Committee on Immunization Practices (ACIP) ≥2 weeks before the time of specimen collection, (i.e., administration of DTaP at ages 2, 4, and 6 months, with the 4th and 5th booster doses administered at ages 15–18 months and 4–6 years, respectively) (*4*). Doses administered at less than the recommended interval since the previous dose were not considered valid and were not counted. Participants who had received 1 or 2 valid DTaP doses were considered partially vaccinated. Participants with no recorded doses were considered unvaccinated.

### Statistical Analysis

Demographic and vaccination characteristics were compared between case-patients and control patients using Fisher’s exact tests for variables with two categories and Pearson’s chi-square tests for those with more than two categories, with p-values <0.05 considered statistically significant. Because case-patients and control patients were matched by birth year and health care facility, and because race was considered potentially related to facility use, as many Alaska Native persons seek care at Tribal facilities, p-values for age and race were not calculated. Conditional logistic regression models were used to calculate crude and adjusted odds ratios (ORs) for pertussis disease in unvaccinated and partially vaccinated persons relative to fully vaccinated participants, accounting for matching factors (facility and birth year) and adjusting for the a priori identified covariates of race and calendar month of infection. Vaccine effectiveness (VE) was calculated as (1 − OR) × 100%. Trends in time since vaccination among fully vaccinated children were evaluated using Cochran-Armitage (categorical elapsed time) and Wilcoxon tests (continuous elapsed time). A sensitivity analysis was also performed to assess whether facility matching substantially influenced VE estimates. All analyses were performed using R (version 4.4.1; R Foundation). This activity was reviewed by CDC, considered not research, and conducted consistent with applicable federal law and CDC policy.*

## Results

### Characteristics of Study Participants

During May 1–November 30, 2024, a total of 113 laboratory-confirmed pertussis cases among children aged 6 months–6 years was reported to the Alaska Section of Epidemiology (incidence = 193.0 cases per 100,000 children). Seven (6.2%) children with pertussis who were born outside Alaska, and 22 (19.5%) for whom no age-matched controls were available at the reporting facility were excluded, leaving 84 (79%) patients with laboratory-confirmed pertussis. These 84 case-patients and 229 matched control patients (2.7 controls per case-patient) from 17 facilities across Alaska were included in the analysis (Table 1). The median participant age was 2.2 years (IQR = 1.0–4.2 years). Case-patients were significantly more likely to be female (59.5%; 50) than were control patients (42.4%; 97) and were less likely to be eligible for the Vaccines for Children program (22.6%; 19 versus 41.9%; 96); no statistically significant difference was observed by ethnicity.

**TABLE 1:** Demographic and vaccination status of patients in case-control analysis of diphtheria and tetanus toxoids and acellular pertussis vaccine effectiveness — Alaska, May–November 2024

Characteristic	No. (%) N = 313		p-value
	Case-patients* n = 84	Control patients^†^ n = 229	
**Median age, yrs (IQR)**	2.0 (1.0–3.5)	2.2 (1.1–3.7)	NA^§^
**Age group**			
6 mos–2 yrs	41 (48.8)	106 (46.3)	NA^§^
3–6 yrs	43 (51.2)	123 (53.7)	—
**Sex**			
Female	50 (59.5)	97 (42.4)	0.005^¶^
Male	33 (39.3)	132 (57.6)	—
**Race**			
American Indian or Alaska Native	32 (38.1)	94 (41.0)	NA^§^
Asian or Pacific Islander	3 (3.6)	22 (9.6)	—
Black or African American	1 (1.2)	7 (3.1)	—
White	34 (40.5)	61 (26.6)	—
Other or multiracial	14 (16.7)	45 (19.7)	—
**Ethnicity**			
Hispanic or Latino	0 (—)	9 (3.9)	0.05**
Not Hispanic or Latino	55 (65.5)	163 (71.2)	—
Unknown	29 (34.5)	57 (24.9)	—
**DTaP vaccination status**			
Fully vaccinated^††^	21 (25.0)	177 (77.3)	<0.001^¶^
Partially vaccinated^§§^	11 (13.1)	24 (10.5)	—
Unvaccinated^¶¶^	52 (61.9)	28 (12.2)	—
VFC eligible***			
Yes	19 (22.6)	96 (41.9)	<0.001^¶^
No	13 (15.5)	103 (45.0)	—
Unknown	52 (61.9)	30 (13.1)	—

**Abbreviations:** DTaP = diphtheria and tetanus toxoids and acellular pertussis vaccine; NA = not applicable; VFC = Vaccines for Children.

* Alaska-born children aged 6 months–6 years with first-time, laboratory-confirmed pertussis reported in Alaska, with symptom onset during May 1–November 30, 2024.

^†^ Alaska-born children aged 6 months–6 years with acute cough who received testing for respiratory viral pathogens and received a negative pertussis test result or did not receive testing for pertussis. Because negative pertussis test results are not reportable in Alaska, it is not known how many control patients received a negative test result and how many did not receive testing for pertussis.

^§^ Case-patients and control patients were matched on birth year and health care facility (race-dependent), and therefore no p-value was calculated.

^¶^ Fisher’s exact test p-value.

** Pearson’s chi-square test p-value.

^††^ Documented receipt of ≥3 valid DTaP doses ≥2 weeks before specimen collection.

^§§^ Documented receipt of 1 or 2 valid DTaP doses ≥2 weeks before specimen collection.

^¶¶^ No documented receipt of any DTaP dose.

*** VFC provides vaccines to uninsured or underinsured children.

### Vaccination Status of Participants

Among all 313 participants, 198 (63%) were fully vaccinated, 35 (11%) were partially vaccinated, and 80 (26%) were unvaccinated. Case-patients were significantly more likely to be unvaccinated (61.9%; 52) than were control patients (12.2%; 28) and less likely to be fully vaccinated (25.0%; 21) than were control patients (77.3%; 177).

### Vaccine Effectiveness

Compared with unvaccinated children, adjusted VE was 96.2% among fully vaccinated participants and 85.3% among those who were partially vaccinated (Table 2). The odds of laboratory-confirmed pertussis among unvaccinated children were approximately 26 times as high as than those among fully vaccinated children (adjusted OR 26.1; 95% CI = 9.6–70.9). Partially vaccinated children had 3.8 times the odds of laboratory-confirmed pertussis compared with fully vaccinated children (adjusted OR = 3.8). A sensitivity analysis performed without facility matching produced similar unadjusted VE estimates (Supplementary Table 1) (Supplementary Table 2). Among fully vaccinated participants, interval since receipt of the last pertussis vaccine dose did not statistically significantly differ between case-patients and control patients (Table 3).

**TABLE 2:** Odds ratios for pertussis disease associated with diphtheria and tetanus toxoids and acellular pertussis vaccination and estimated vaccine effectiveness — Alaska, May–November 2024

Characteristic	DTaP vaccination status		
	Fully vaccinated*	Partially vaccinated^†^	Unvaccinated^§^
**Study group^¶^**			
Case-patients (n = 84), no. (%)	21 (25.0)	11 (13.1)	52 (61.9)
Control patients (n = 229), no. (%)	177 (77.3)	24 (10.5)	28 (12.2)
**OR (95% CI) for pertussis disease relative to unvaccinated children**			
Unadjusted	0.1 (0–0.1)	0.2 (0.1–0.5)	1 (Ref)
Adjusted**	0 (0–0.1)	0.2 (0.1–0.4)	1 (Ref)
**OR (95% CI)**^††^ **for pertussis disease relative to fully vaccinated children**			
Unadjusted	1 (Ref)	4.2 (1.8–9.8)	21.6 (8.9–52.5)
Adjusted**	1 (Ref)	3.8 (1.7–8.6)	26.1 (9.6–70.9)
**Pertussis vaccine effectiveness, % (95% CI)**			
Unadjusted	95.4 (88.7–98.1)	80.6 (55.1–91.6)	1 (Ref)
Adjusted**	96.2 (89.6–98.6)	85.3 (64.4–94.0)	1 (Ref)

**Abbreviations**: DTaP = diphtheria and tetanus toxoids and acellular pertussis vaccine; OR = odds ratio; Ref = referent group.

* Documented receipt of ≥3 valid DTaP doses ≥2 weeks before specimen collection.

^†^ Documented receipt of 1 or 2 valid DTaP doses ≥2 weeks before specimen collection.

^§^ No documented receipt of any DTaP dose.

^¶^ Patients were matched by birth year and facility.

** Adjusted for patient race, sex, and calendar month.

^††^ Presented for ease of comparison.

**TABLE 3:** Characteristics of case-patients and control patients who were fully vaccinated* against pertussis, by time since receipt of most recent pertussis-containing vaccine dose — Alaska, May–November 2024

Characteristic	No. (%)			p-value
	Case-patients n = 21	Control patients n = 177	Total N = 198	
Median age, yrs (IQR)	2.9 (1.3–5.5)	2.6 (1.2–5.2)	**2.7 (1.2–5.3)**	0.72^†^
**Time since last DTaP dose**				
Months, median (IQR)	15.3 (7.6–23.6)	10.2 (4.2–19.2)	**10.9 (4.4–20.1)**	0.23^†^
15–364 days	9 (42.9)	95 (53.7)	**104 (52.5)**	0.19^§^
1–2 yrs	7 (33.3)	51 (28.8)	**58 (29.3)**	—
≥3 yrs	5 (23.8)	31 (17.5)	**36 (18.2)**	—

**Abbreviation**: DTaP = diphtheria and tetanus toxoids and acellular pertussis vaccine.

* Received ≥3 doses of DTaP vaccine ≥2 weeks before specimen collection.

^†^ Wilcoxon test p-value.

^§^ Cochran Armitage test p-value.

## Discussion

In this study of DTaP VE, unvaccinated children in Alaska had approximately 26 times the odds of acquiring laboratory-confirmed pertussis compared with children who had received ≥3 DTaP doses. These real-world findings support childhood vaccination recommendations to protect young children against pertussis. Among children with pertussis, those who are unvaccinated are more likely than are those who are vaccinated to experience severe illness or require hospitalization (*5*).

The VE findings in this report (96.2% for fully vaccinated children and 85.3% for partially vaccinated children aged 6 months–6 years) are higher than those found in prelicensure studies (*1*) but consistent with other real-world studies (*6*–*8*). This finding is likely because of the focus on laboratory-confirmed cases and on younger children. Unlike cases in a test-negative prelicensure study design, cases among children with milder symptoms who did not seek health care would not appear in these data; however, these cases are likely of less clinical significance.

The longer interval since receipt of the most recent vaccine dose among fully vaccinated case-patients compared with control patients, although not statistically significant in this analysis, aligns with existing research on waning of vaccine-induced immunity (*3*,*9*). Waning immunity, including among participants who had received 3 doses but had not yet received the recommended 4th and 5th booster doses recommended for children aged 15–18 months and 4–6 years, respectively, might have played a role in susceptibility to pertussis during the outbreak. Future longitudinal studies could further examine the role of waning of vaccine-induced immunity.

This analysis has several important strengths, including the matched case-control design, which minimized selection bias and allowed for more accurate estimation of VE. Matching by health care facility helped control for variations in patient demographic characteristics, health care access, local circulation of pertussis, and minimized bias arising from differences across facilities in testing protocols and diagnostic capabilities (e.g., test sensitivity and specificity). In Alaska, because certain large facilities serve almost exclusively Alaska Native patients, who have substantially higher vaccination rates than do other major racial and ethnic groups in Alaska, matching by facility helped to ensure that comparisons were made between case-patients and control patients with comparable health care access and health behaviors. The similarly high unadjusted VE estimates with and without facility matching suggests minimal impact of excluded cases on results.

### Limitations

The findings in this report are subject to at least seven limitations. First, because case-patients and control patients were identified through public health surveillance data, symptom severity among case-patients and control patients was not available for comparison and therefore was not assessed. In addition, because case-patients were not included if at least one age-matched control patient was not available, this analysis might not represent patients attending the smallest health care facilities. Second, because control patients were children who received testing during the same period for respiratory viral pathogens but who received a negative pertussis test result or did not receive testing for pertussis, control patients might include persons with pertussis who did not receive testing, resulting in misclassification. Because this misclassification could occur with fully vaccinated, partially vaccinated, or unvaccinated control patients, it would likely be nondifferential. Third, maternal pertussis vaccination during pregnancy, which might provide some immunity to the youngest participants (*10*), was not assessed and might have influenced pertussis rates among younger infants; this effect would be expected to be small because passive maternal antibody protection wanes by age 8 months (*10*). Fourth, whereas analysis was restricted to children born in Alaska, vaccination status might have been misclassified for participants who were vaccinated in another state. This misclassification could occur with both case-patients and control patients and is thus unlikely to substantially bias VE estimates. Fifth, the CI for the OR for pertussis disease in unvaccinated versus fully vaccinated children is relatively wide, and a larger sample size might have permitted a more precise estimation of OR; however, even the lowest bound of the 95% CI reflects approximately a tenfold difference in odds. Sixth, sample size did not permit additional subgroup analysis by interval since receipt of the most recent vaccine dose or by number of doses among those who were fully vaccinated (e.g., receipt of 3 versus 4 or 5 doses); a larger sample size might have permitted more detailed analyses of subcategories. Finally, the results describe laboratory-confirmed cases among children aged 6 months–6 years and might not be generalizable to older children or milder cases among children who did not require medical attention.

### Implications for Public Health Practice

These findings demonstrate that pertussis vaccination was highly effective at preventing laboratory-confirmed pertussis during a large statewide outbreak. Declining childhood pertussis vaccination coverage in Alaska might have contributed to the size of the 2024 outbreak. These findings reinforce 2018 ACIP recommendations for pertussis vaccination of all infants, beginning at age 2 months.

## Footnotes

* 45 C.F.R. part 46.102(l), 21 C.F.R. part 56; 42 U.S.C. Sect. 241(d); 5 U.S.C. Sect. 552a; 44 U.S.C. Sect. 3501 et seq.
